# Transmissible cancers, are they more common than thought?

**DOI:** 10.1111/eva.12372

**Published:** 2016-05-17

**Authors:** Beata Ujvari, Robert A. Gatenby, Frédéric Thomas

**Affiliations:** ^1^ Centre for Integrative EcologySchool of Life and Environmental SciencesDeakin UniversityWaurn PondsAustralia; ^2^ Department of RadiologyH. Lee Moffitt Cancer Center and Research InstituteTampaFLUSA; ^3^ Centre for Ecological and Evolutionary Research on Cancer (CREEC)UMR CNRS/IRD/UM1 MIVEGECMontpellierFrance

**Keywords:** ecology, evolution, Tasmanian devil, transmissible cancer

Although many cancers are associated with infectious agents (Ewald and Swain Ewald 2015), only four naturally occurring transmissible cancers have so far been identified in dogs, soft‐shell clams and Tasmanian devils (DFT1 and DFT2) (Pye et al. 2015). The recent discovery of DFT2 (Pye et al. 2015) provides an intriguing story to the evolution of transmissible cancers and poses several questions:

## How could two transmissible cancers emerge in the same species?

Cancer cell transmission to a new host is, like the metastatic cascade, a complex multistep process, with distinct micro‐ and macro‐environmental barriers (Gatenby and Gillies 2008). A crucial step of cancer cell transmission requires the tumour cells to overcome histocompatibility barriers, which is highly facilitated by the reduced genetic diversity of devils. Furthermore, DFTD has originated form peripheral nerve cells that possess the astonishing capacity to reverse functional and developmental commitments (Masaki et al. 2013). The combination of a permissive host micro‐environment and highly plastic cells of origin could have provided several cells to sabotage multicellularity, enter a selfish lifestyle and become transmissible malignant cell lineages.

## How will the two DFT variants evolve?

It seems unlikely that DFT2 arose from DFT1 even though the temporal sequence of their discovery suggests it. That is, it is difficult to imagine that the chromosome fragments in DFT1 coalescing into the relatively normal DFT2 karyotype. The other direction (i.e. DFT2 evolving into DFT1) seems plausible, or most likely, the two diseases have arose separately.

An important question is whether the DFT2 tumour is slower growing than DFT1. As the latter seems to grow very quickly, rapidly kill the animals and decimate the host population, the time of potential transmission is short. In this setting, a slower growing tumour may be fitter as the slower growth permits a longer time for transmission in an increasingly sparse population. It will be interesting to see whether slower growing phenotypes become more prevalent as the population steadily declines (Ujvari et al. 2014). The epidemic may actually select for less aggressive tumour phenotypes, and DFTD perhaps will ultimately evolve into a relatively benign tumour. Importantly, according to the speciation theory of cancer evolution, cancers, particularly transmissible ones, could represent new cellular species (Duesberg et al. 2011).

## Are transmissible cancers rare?

We propose that the emergence of transmissible cancers requires a ‘perfect storm’ with the rare confluence of multiple host and tumour cell traits (Ujvari et al. 2016). At least four key factors are required: (i) shedding of tumour cells from the affected host, (ii) survival of tumour cells during the host–host transit, (iii) a permissive environment facilitating invasion and (iv) adaptation to novel habitats and evasion of immune attacks in the foreign host. While this rare confluence of traits explains the rarity of tumour cell transmission, it also suggests that when it eventuates, multiple emergences can theoretically happen as long as the favourable window persists. It is possible that during the eons of evolution, several contagious cancers have evolved, but due to their detrimental impact on host fitness, selection has eliminated them, as well as they might have driven their hosts to extinctions. Consequently, it is conceivable that due to our limited perception of the evolutionary timescale, we fail to recognize extinct contagious cancers and hence erroneously identify them to be rare.
